# Heart-rate-variability (HRV), predicts outcomes in COVID-19

**DOI:** 10.1371/journal.pone.0258841

**Published:** 2021-10-28

**Authors:** Maartje B. A. Mol, Maud T. A. Strous, Frits H. M. van Osch, F. Jeroen Vogelaar, Dennis G. Barten, Moshe Farchi, Norbert A. Foudraine, Yori Gidron

**Affiliations:** 1 Department of Surgery, VieCuri Medical Centre Venlo, Venlo, The Netherlands; 2 Department of Intensive Care, VieCuri Medical Centre Venlo, Venlo, The Netherlands; 3 Department of Epidemiology, VieCuri Medical Centre Venlo, Venlo, The Netherlands; 4 Department of Emergency Medicine, VieCuri Medical Centre Venlo, Venlo, The Netherlands; 5 School of Social Work, Tel-Hai College, Qiryat Shemona, Israël; 6 Faculty of Welfare and Health Sciences, University of Haifa, Haifa, Israel; University of Minnesota, UNITED STATES

## Abstract

**Background:**

Patients with COVID-19 present with a variety of clinical manifestations, ranging from mild or asymptomatic disease to severe illness and death. Whilst previous studies have clarified these and several other aspects of COVID-19, one of the ongoing challenges regarding COVID-19 is to determine which patients are at risk of adverse outcomes of COVID-19 infection. It is hypothesized that this is the result of insufficient inhibition of the immune response, with the vagus nerve being an important neuro-immuno-modulator of inflammation. Vagus nerve activity can be non-invasively indexed by heart-rate-variability (HRV). Therefore, we aimed to assess the prognostic value of HRV, as a surrogate marker for vagus nerve activity, in predicting mortality and intensive care unit (ICU) referral, in patients hospitalized with COVID-19.

**Methods:**

A retrospective cohort study including all consecutive patients (n = 271) diagnosed and hospitalized with COVID-19 between March 2020 and May 2020, without a history of cardiac arrhythmias (including atrial and ventricular premature contractions), pacemaker, or current bradycardia (heart rate <50 bpm) or tachycardia (heart rate >110 bpm). HRV was based on one 10s ECG recorded at admission. 3-week survival and ICU referral were examined.

**Results:**

HRV indexed as standard deviation of normal to normal heartbeat intervals (SDNN) predicted survival (H.R. = 0.53 95%CI: 0.31–0.92). This protective role was observed only in patients aged 70 years and older, not in younger patients. HRV below median value also predicted ICU referral within the first week of hospitalization (H.R = 0.51, 95%CI: 0.29–0.90, P = 0.021).

**Conclusion:**

Higher HRV predicts greater chances of survival, especially in patients aged 70 years and older with COVID-19, independent of major prognostic factors. Low HRV predicts ICU indication and admission in the first week after hospitalization.

## Introduction

The severe acute respiratory syndrome due to coronavirus 2 (SARS-CoV-2), which causes coronavirus disease 2019 (COVID-19), has been declared a pandemic by the World Health Organization [1]. As of 27.01.2021, 99,638,507 confirmed cases of the coronavirus are officially reported globally in over 200 countries, including 2,141,468 deaths [2]. Patients with COVID-19 present with a variety of clinical manifestations, which most frequently consist of flu-like symptoms, ranging from mild or asymptomatic disease to severe illness and death [3–6]. The clinical manifestations of COVID-19 may be a consequence of the variable intensity of the immune response [5]. Whilst previous studies have clarified these and several other aspects of COVID-19, one of the ongoing challenges regarding COVID-19 is to determine which patients are at risk of adverse outcomes of COVID-19 infection.

During infection with the coronavirus, the inflammatory pathway is induced by the virus, whereupon the sympathetic nervous system is activated and an inflammatory response is elicited [7]. An important neuro-immuno-modulator in the inflammatory pathway is the vagus nerve. The vagus informs the brain about peripheral inflammation and then reflexively inhibits this inflammation [8]. This inhibition is established by activation of the hypothalamic-pituitary-adrenal axis, which suppresses inflammation by producing cortisol. In addition, a descending efferent vagal-to-sympathetic neural conversion occurs at the celiac ganglion and reaches the spleen, where a sub-class of T-cells secrete acetylcholine and inhibit splenic macrophages from producing inflammatory cytokines [9–11]. When this anti-inflammatory regulatory response works properly, it limits the dissemination of viral infections and is vital for the control and resolution of the infection and its inflammatory consequences. But, in cases where vagus activity is low, the inflammatory response can be left uncontrolled, contributing to hyper-inflammation–the so-called “cytokine storm” [12]. In addition, during induced infection, vagus nerve stimulation is associated with increased natural killer activity and CD8+ cells [13]. Thus, the vagus nerve controls inflammation on one hand, but can increase anti-viral immunity on the other hand.

The activity of the vagus nerve can be indexed by the measurement of heart-rate-variability (HRV). In sinus rhythm, HRV reflects time fluctuations in the intervals between normal consecutive heartbeats and reflects efferent vagus nerve activity to the heart [14–16]. Though a recent study did not show a correlation between vagal activity and HRV [17], an older study found a profound correlation (R = 0.88) between actual vagus nerve activity and HRV, and that HRV changed in response to vagomimetic medication [18]. Furthermore, HRV, especially the high-frequency parameters, is considered an index of vagal activity [19]. Hence, by measuring a patients’ HRV, for instance on an ECG, vagus nerve activity can be indexed [20]. Two studies examined the role of HRV in Covid-19. One study found in 17 Covid-19 patients that low HRV preceded increases in the inflammatory marker C-reactive protein [21]. Another study performed on 14 Covid-19 patients admitted to the intensive care unit (ICU) found that while HF-HRV (a purely vagal index) was higher in patients who later died, the HRV time domain parameter of SDNN (reflecting mostly vagal but also partly sympathetic activity) was lower in those who subsequently died [22], However, these studies used small sample sizes and did not statistically adjust for important confounders such as age and comorbidities.

The purpose of this study was to assess the prognostic value of HRV as a surrogate marker for vagus nerve activity in predicting mortality in patients hospitalized with COVID-19, independent of known prognostic factors. In addition, we examined whether HRV was discriminative for ICU referral during the admission. Finally, we also tested whether vagus nerve activity moderated (weakened) effects of other prognostic factors on mortality.

## Method

### Study population

The study population included all consecutive patients diagnosed with COVID-19 (defined as positive PCR assay) admitted to our hospital between March 2020 and May 2020 at VieCuri Medical Centre, Venlo, the Netherlands. Patients with cardiac arrhythmias (including atrial and ventricular premature contractions), pacemakers, bradycardia (heart rate <50 bpm), or tachycardia (heart rate >110 bpm) were excluded as these precluded a reliable calculation of the HRV. In case no ECG was obtained during admission, calculation of the HRV was impossible and the patient had to be excluded from the study.

### Design and data collection

Data were retrospectively collected from patients’ medical records based on the WHO-COVID-case record form [23]. However, HRV was measured at the time of admission, and the primary outcomes (ICU admission and death) were observed later. This encompassed information on patient characteristics including sex, age, BMI, comorbidities (chronic heart disease including all heart diseases except hypertension, diabetes, hypertension, obesity, among others), COVID-symptoms, diagnostics (COVID-PCR, ECG, laboratory results, microbiology, and radiological results), medication, ICU-admission, length of stay and outcome. For the present study, we focused on survival at three weeks from admission. This study was approved by the research committee and the Board of Directors of VieCuri Medical Centre. Due to the retrospective and observational approach of the study, a waiver of informed consent was provided.

### Heart rate variability

Heart rate variability was analyzed using one 12-lead 10-second ECG (150Hz) recording per patient, obtained during hospital admission. In the case of having multiple ECGs taken on the day of admission, the ECG with the best quality was used for HRV-analyses. In case no ECG was taken on the day of admission, the ECG that was made after presentation with COVID-related symptoms, closest to the day of admission, was used for HRV-analyses.

The time between two consecutive R-peaks was measured in lead II with an accuracy of 0.2ms, using the MUSE^TM^-ECG system (General Electric Company, Massachusetts, United States). HRV was presented by the time-domain HRV parameters of the standard deviation of NN-intervals (SDNN) and the root mean square of successive differences (RMSSD) in milliseconds, calculated using common formulas [24]:

$$SDNN=\sqrt{\frac{1}{n-1}\sum_{i=1}^{n}({RR}_{i}-{RR}_{mean}{)}^{2}}$$ (1)

$$RMSSD=\sqrt{\frac{1}{n-1}\sum_{i=1}^{n-1}({RR}_{i+1}-{RR}_{i}{)}^{2}}$$ (2)

SDNN and RMSSD obtained from 10s ECGs were found to correlate with results of ECGs of longer durations [25]. Furthermore, there is a strong correlation between HRV from 10sec and 5min ECGs [26]. The power spectral analysis HRV parameters of low frequency (LF) and high frequency (HF) HRV can only be obtained from longer ECG recordings and were therefore not implementable in this study [24, 27, 28]. The cut-off point for dividing HRV parameters into two groups (low vs high) was 8, based on the median SDNN and RMSSD in the study population.

### Study outcomes

The primary endpoint of this study was overall survival at three weeks from admission. Survival was defined as alive or dead, alive being defined as discharged from the hospital and dead being defined as the death of the patient during hospitalization. In addition, we examined whether HRV was discriminative for intensive care unit (ICU) referral during the admission, after seven days and three weeks. Several patients had a medical indication for ICU admission but were not admitted because of treatment restrictions based on age, comorbidities, and functional capacity before admission to the hospital. Consequently, some of these patients died at the hospital ward when in need of intensive care. For this reason, we also examined whether HRV was associated with an indication for ICU admission. Patients who were not transferred to the ICU because of treatment restrictions, but died at the hospital ward within seven days after hospitalization were labeled as having an ICU indication, as were the others who were admitted to the ICU in this period.

### Statistical analysis

We utilized descriptive statistics to provide an overview of confounding and main study variables of the study population (patient characteristics, including age, sex, comorbidities, HRV and outcomes). Because of normal distribution the continuous variable age was compared between HRV-groups using unpaired t-test. All other variables were categorical and were compared between HRV-groups using chi-square statistics.

The association between survival and ICU-indication, with HRV was tested using multivariate logistic regression. For survival and ICU-admission this was followed by multivariate cox regression. The confounders in the multivariate regression were included according to the following criteria: those which were significantly univariately predictive of the analyzed endpoint in the present study and/or which were found in a recent meta-analysis to predict survival in COVID-19 [6]. Since age strongly predicts survival in COVID-19 and since HRV declines with age [6, 29], we re-tested the multivariate logistic regression in people below and above age 70, the approximate median age in this sample. Data analysis was performed using IBM SPSS statistics, version 24.0. A two-tailed p-value ≤ 0.05 was considered significant in all analyses.

## Results

### Basic characteristics

The mean age of the study population was 68 years (range 25 to 95). Patients’ mean SDNN was considerably lower than 50ms and their mean RMSSD was lower than 42, norms in the healthy population [19]. Approximately 60% of the sample were men, a third were obese, half had hypertension, and three-week in-hospital mortality was nearly 22%, as shown in Table 1.

**Table 1:** Descriptive data of included patients according to heart-rate-variability (HRV).

Variables	SDNN ≤ 8	SDNN > 8	p	RMSSD ≤ 8	RMSSD > 8	p
N	142	129		147	124	
Age, years*	68 [59–75]	71 [60–79]	0.106	66 [57–73]	72 [62–81]	<0.001
Sex, n (%)			0.874			0.508
Women	57 (40.1)	53 (41.1)		57 (38.8)	53 (42.7)	
Men	85 (59.9)	76 (58.9)		90 (61.2)	71 (57.3)	
Comorbidities, n (%)						
Chronic heart disease	37 (27.6)	36 (30.3)	0.644	28 (20.0)	45 (39.8)	0.001
Hypertension	70 (51.1)	62 (49.6)	0.809	63 (44.4)	69 (55.6)	0.034
Obesity	50 (37.0)	34 (27.6)	0.108	53 (37.6)	31 (26.5)	0.058
Diabetes	21 (16.9)	20 (17.7)	0.877	24 (17.9)	17 (16.5)	0.777

* Non normal-distributed data presented as median with interquartile range.

Note: SDNN = standard deviation of normal to normal heartbeat intervals; RMSSD = root mean square of successive differences between adjacent heartbeats. Both SDNN and RMSSD cut-offs are in msec.

In 13 patients, no ECG was taken on the day of admission. Of those, all had an ECG made within three days after admission, which was used for HRV analysis.

### Three weeks in-hospital survival

In multivariate analysis HRV (indexed as SDNN) showed to be a significant predictor of survival, independent of age and chronic heart disease (odds ratio (O.R.) and 95% confidence interval (95%CI): O.R. = 0.51, 95%CI: 0.27–0.97, p < 0.05), as shown in Table 2. This analysis was then followed by a multivariate Cox regression, taking into account time until the event (alive or dead). Here too, HRV (indexed as SDNN) significantly and independently predicted survival within three weeks post-admission, as shown in Table 3. Patients with a higher HRV (indexed as SDNN > 8) were at a significantly lower risk of death than those with lower HRV. RMSSD was not a significant predictor of survival, independent of age and chronic heart disease, as analyzed in multivariate logistic regression and cox regression, as shown in Tables 2 and 3.

**Table 2:** Odds ratio for mortality within three weeks after admission using logistic regression.

	Logistic regression				Logistic regression		
	Unadjusted	Model 1	Model 2		Unadjusted	Model 1	Model 2
SDNN				RMSSD			
≤8	reference	reference	reference	≤8	reference	reference	reference
>8	0.64 (0.35–0.17)	0.51 (0.27–0.96)	0.51 (0.27–097)	>8	0.96 (0.53–1.74)	0.58 (0.30–1.13)	0.55 (0.28–1.07)

Model 1 adjusted for age.

Model 2 adjusted for age and chronic heart disease.

Note: SDNN = standard deviation of normal to normal heartbeat intervals; RMSSD = root mean square of successive differences between adjacent heartbeats. Both SDNN and RMSSD cut-offs are in msec.

**Table 3:** Hazard ratio for mortality within three weeks after admission using Cox-regression.

	Cox regression				Cox regression		
	Unadjusted	Model 1	Model 2		Unadjusted	Model 1	Model 2
SDNN				RMSSD			
≤8	reference	reference	reference	≤8	reference	reference	reference
>8	0.70 (0.41–1.19)	0.53 (0.31–0.92)	0.55 (0.32–0.95)	>8	0.99 (0.59–1.67)	0.63 (0.36–1.11)	0.61 (0.35–1.07)

Model 1 adjusted for age.

Model 2 adjusted for age and chronic heart disease.

Note: SDNN = standard deviation of normal to normal heartbeat intervals; RMSSD = root mean square of successive differences between adjacent heartbeats. Both SDNN and RMSSD cut-offs are in msec.

### Survival in older patients

**Fig 1:**
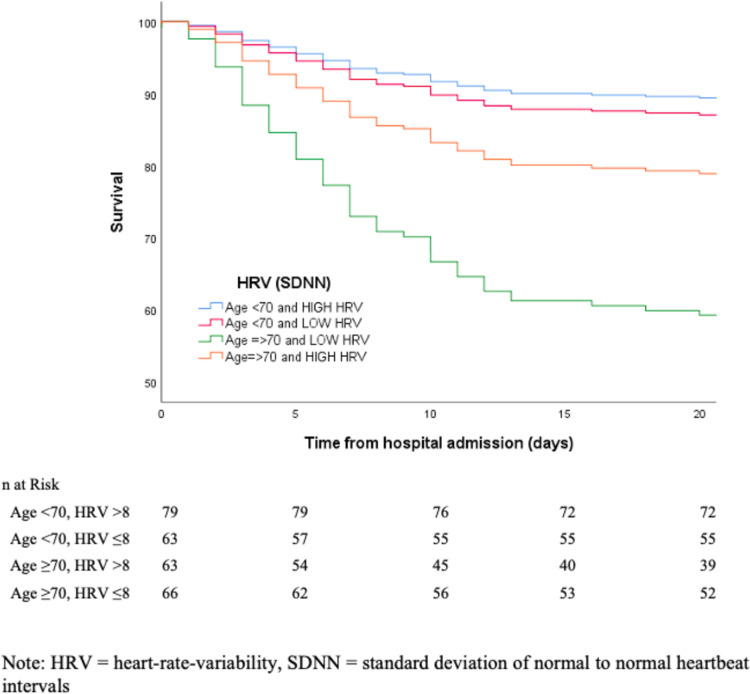
Effects of age on mortality from COVID-19 as a function of vagal nerve activity (HRV).

In this analysis, HRV did not significantly predict survival at three weeks post-admission in patients below age 70, independent of confounders (O.R = 1.12, 95%CI: 0.38–3.30, p > 0.05). In contrast, HRV strongly predicted survival in patients ≥70 years old, independent of confounders (O.R = 0.28, 95%CI: 0.12–0.66, p < 0.005). Similarly, using a Cox regression, SDNN significantly predicted survival within three weeks post-admission only in patients aged 70 years and older, independent of confounders (H.R = 0.32, 95%CI: 0.16–0.64, p = 0.001) but not in those younger than age 70 (H.R = 1.28, 95%CI: 0.48–3.44, p > 0.05). This result is depicted in Fig 1.

### Moderating effect of HRV on risk factors and their association with mortality

Since age had the strongest effect on survival, we examined the moderating effect of HRV on this relationship. Indeed, older age (≥70) predicted a higher risk of death than younger age, but only in patients with lower HRV (X^2^(1) = 19.30, p < 0.001), but not in patients with higher HRV (X^2^(1) = 1.65, p > 0.05).

### HRV and ICU admission

In multivariate Cox regression analysis, HRV (RMSSD) was a significant predictor of ICU admission, independent of age and chronic heart disease. Patients with HRV (RMSSD) higher than 8 were at lower risk of being transferred later from the hospital ward to ICU (H.R = 0.51, 95%CI: 0.29–0.90, p = 0.021), as shown in Table 4. All patients admitted to the ICU were admitted within seven days from hospital admission. However, not all patients that had an ICU indication were admitted to the ICU. Some of them died at the hospital ward because of treatment restrictions agreed based on older age, comorbidities, and functional capacity before admission to the hospital. In a multivariate logistic regression analysis, HRV (SDNN and RMSSD) was a significant predictor of indication for ICU admission within seven days after hospitalization. Patients with relatively higher HRV (SDNN and RMSSD) were at lower risk of needing ICU-care (SDNN; O.R. = 0.59, 95%CI: 0.35–0.99, p = 0.048; RMSSD; O.R. = 0.53, 95%CI: 0.30–0.93, p = 0.027), as shown in Table 5.

**Table 4:** Hazard ratio for ICU admission within three weeks after admission.

	Cox regression				Cox regression		
	Unadjusted	Model 1	Model 2		Unadjusted	Model 1	Model 2
SDNN				RMSSD			
≤ 8	reference	reference	reference	≤ 8	reference	reference	reference
> 8	0.62 (0.37–1.03)	0.65 (0.39–1.09)	0.65 (0.39–1.08)	> 8	0.46 (0.27–0.80)	0.51 (0.29–0.90)	0.51 (0.29–0.90)

Model 1 adjusted for age.

Model 2 adjusted for age and cardiac disease.

Note: ICU = intensive care unit, SDNN = standard deviation of normal to normal heartbeat intervals, RMSSD = root mean square of successive differences between adjacent heartbeats. Both SDNN and RMSSD cut-offs are in msec.

**Table 5:** Odds ratio for indication for ICU admission within seven days after hospital admission.

	Logistic regression				Logistic regression		
	Unadjusted	Model 1	Model 2		Unadjusted	Model 1	Model 2
SDNN				RMSSD			
≤ 8	reference	reference	reference	≤ 8	reference	reference	reference
> 8	0.60 (0.36–1.01)	0.59 (0.35–0.99)	0.59 (0.35–0.99)	> 8	0.60 (0.35–1.02)	0.56 (0.32–097)	0.53 (0.30–0.93)

Model 1 adjusted for age.

Model 2 adjusted for age and cardiac disease.

Note: ICU = intensive care unit, SDNN = standard deviation of normal to normal heartbeat intervals, RMSSD = root mean square of successive differences between adjacent heartbeats. Both SDNN and RMSSD cut-offs are in msec.

## Discussion

To our knowledge, this is among the first studies that examined the prognostic role of HRV as a surrogate marker for vagus nerve activity in COVID-19. Our results revealed that HRV predicted survival in the first three weeks after hospitalization, independent of known other prognostic factors like age and heart disease. In patients aged 70 and older, HRV above 8 predicted a 3.1 times higher chance to survive when compared to lower HRV, independent of confounders. This relationship was not significant in the younger patients. Furthermore, previous studies have shown that age strongly predicts survival in COVID-19, but in the present study, we found that this was only confirmed when HRV was low, suggesting a moderating and protective role of vagus nerve activity in COVID-19 [6].

This COVID-19 pandemic not only challenged us with an unknown disease but also caused major hospital capacity issues, ICU resource shortages in particular. Treatment restrictions as a no-ICU-policy were agreed upon based on age, comorbidities, and functional capacity before admission to the hospital, but could have led to a reduction in the numbers of surviving patients, especially in the first days after admission. Therefore, we not only examined whether HRV predicted actual admission to the ICU, but also ICU indication, when there were no restrictions, by labeling all patients dying within one week after hospitalization as a patient with an ICU indication. In both scenarios, HRV predicted actual or indicated admission to the ICU.

The median level of HRV (SDNN = 8msec) is in line with past studies on the effects of sepsis on HRV [30], and may reflect an elevated sympathetic response during such a life-threatening illness.

The results observed here support those of Aragon-Benedi et al. (2021) who found that lower SDNN predicted mortality in Covid-19 patients in the ICU, but contradict their findings that high HF-HRV (a purely vagal index) predicted higher chance of death in these patients [22]. However, their study was done on patients in the ICU and included a very small sample. In contrast, the present study included a much larger sample, representative of a wider extent of severity of Covid-19 illness.

A fast and well-coordinated innate immune response is the first line of defense against viral infections such as SARS-CoV-2 [31]. After infection with the virus, an incubation period follows, in which a specific adaptive immune response is required to eliminate the virus and to preclude disease progression [32]. However, in case of an overactive immune response, excessive inflammation will follow, leading to severe stages and complications from COVID-19 [31]. In patients with severe stage COVID-19, changes in laboratory parameters including elevated serum cytokine and chemokine levels have been found, suggesting that a “cytokine storm” was induced. This hyper-inflammatory response leads to a severe progression of the disease [33]. It was found that it is the innate inflammation in the lungs, induced by the SARS-CoV-2 infection and largely mediated by pro-inflammatory macrophages and granulocytes, which leads to the development of acute respiratory distress syndrome (ARDS), a life-threatening respiratory disorder [32].

The inflammatory response following the incubation with SARS-CoV-2 is partly mediated by the sympathetic nervous system [15]. However, it is the vagus nerve that informs the brain about peripheral inflammation via its interleukin-1 receptors, and then, when well-functioning, the vagus nerve reflexively inhibits this inflammation. This inhibition includes activation of the hypothalamic-pituitary-adrenal axis, which produces cortisol that suppresses inflammation. In addition, this inhibition includes an efferent vagal route via conversion to a sympathetic signal entering the spleen, which causes T-cells to produce acetylcholine. The latter binds to macrophages and suppresses their inflammatory cytokine production [9–11]. The dysregulation of the immune response seen in severe cases of COVID-19, leading to excessive inflammation and ARDS, can thus be the result of impaired vagus nerve activity and impaired regulation of inflammation [8]. By measuring a patients’ HRV, vagus nerve activity can be indexed, and as hypothesized, HRV was found to independently predicted survival in COVID-19.

Previous studies aiming to identify risk factors for infection and disease progression in COVID-19 found an association between age and survival of COVID-19. With age, the effectiveness of the innate and adaptive immune response declines, which results in increased susceptibility to infection, limited repair capacity of damaged cells and tissues, and increased inflammation-related complications [33]. Age has also been correlated with reduced HRV, and thus less vagus nerve regulation of inflammation. Importantly, this study showed that age was a predictor of death in COVID-19 only in case the HRV was relatively low. This shows that the vagus nerve has a moderating and protective role in COVID-19 and when functioning well, the vagus nerve may even weaken the role of aging in prognosis. It could be that more effective vagus neuro-modulation of excessive inflammatory responses was responsible for mitigating the effects of older age on prognosis in COVID-19. However, such mechanisms require verification in future studies.

This study was subject to a number of limitations. The time of study inclusion corresponds to the moment of hospitalization, but the period between onset of symptoms and moment of hospitalization differed between patients, which might have influenced HRV and prognosis. Due to the retrospective design, we did not have full control over the measurement of confounders and on the measurement of HRV. Yet, we tested the effect of and adjusted for several of the studied confounders. Finally, the measurement of HRV only lasted 10 seconds. Nevertheless, past studies have shown that such brief measures predict survival also in cancer [34]. Interestingly, RMSSD did not predict survival, while SDNN did. This is in line with other studies and may be due to the very short ECG of 10sec, not enabling enough variability in the RMSSD parameter [34]. Yet both indexes quite similarly predicted ICU referral. Additionally, while RMSSD reflects vagus nerve activity, SDNN mostly reflects vagus activity but also partly sympathetic activity [35]. This coverage of the full HRV spectrum may explain SDNN’s stronger predictive role in COVID-19. Future studies should further explore this issue.

There were also a number of strengths to this study. To the best of our knowledge, this is among the first studies to empirically link a neuro-immuno-modulatory variable, namely HRV, to the outcome of patients with COVID-19. Given the fact that this disease is a global pandemic, these results may have global public health implications. HRV measurement is easy and non-invasive, and could therefore be easily implemented in daily clinical practice. This study included all consecutive patients admitted to VieCuri Medical Centre, which left us an adequate number of study participants. The database featured a broad spectrum of potential confounders including previously identified prognostic factors in COVID-19, which enabled us to correct for suspected confounding variables.

In conclusion, severe illness and death in COVID-19 are associated with a dysregulated immune response leading to hyper-inflammation, which we believe to be strongly associated with impaired activation of a key neuro-immuno-modulator, the vagus nerve. This study shows that higher HRV predicts greater chances of survival in older patients with COVID-19, independent of major prognostic factors. It also shows that low HRV predicts ICU indication and admission in the first week after hospitalization. Therefore, HRV measurements may aid in the early identification of patients with COVID-19 at risk of clinical deterioration. As impaired vagus nerve activity seems to be associated with hyper-inflammation and ARDS, future studies should focus on the severity of ARDS and ARDS-related survival in relation to vagus nerve activity in patients admitted to the ICU because of COVID-19. These findings have several clinical implications. First, since a 10sec ECG is obtained nearly routinely worldwide, measuring HRV can be used in many countries treating COVID-19 patients. In addition, our and other recent findings can help clinicians to estimate patients’ prognosis and know which patient may need extra medical care [21, 22]. Finally, we suggest to test in an intervention trial the effects of activating the vagus on prognosis of patients with COVID-19. This can be done with medication, or non-invasively with transcutaneous vagal stimulations or if possible for patients, by deep breathing biofeedback. Thus, the effect of non-invasive vagus nerve stimulation, proven to reduce inflammation, may improve COVID-19 survival and needs to be tested in future intervention studies [36].
